# A new approach to enter Retzius space in laparoscopic transabdominal preperitoneal bilateral inguinal hernia repair

**DOI:** 10.1186/s12893-023-01917-8

**Published:** 2023-01-30

**Authors:** Lifei Tian, Le Zhang, Zeyu Li, Likun Yan, Xiaoqiang Wang

**Affiliations:** 1 Department of General Surgery I Section, Shaanxi Provincial People’s Hospital, No. 256 Youyixi Road, Beilin District, Xi’an , 710068 China; 2 Function Testing Lab, Shaanxi Provincial People’s Hospital, Xi’an, 710068 China

**Keywords:** Inguinal hernia, Laparoscope, Retzius space, Bilateral

## Abstract

**Background:**

To investigate the feasibility, safety and efficacy of the right-side approach to enter Retzius space in laparoscopic transabdominal preperitoneal bilateral inguinal hernia repair.

**Methods:**

Retrospective analysis was performed on 189 patients who were diagnosed with bilateral inguinal hernia preoperatively or intraoperatively and underwent selective TAPP in the General Surgery I Section of Shaanxi Provincial People’s Hospital from January 2015 to September 2020. 94 cases were performed using the right-side approach (research group), and 95 cases with conventional approach (control group). Intraoperative and postoperative conditions of the two groups were observed and compared.

**Results:**

All operation were completed successfully. The operative time of research group was significantly shorter than that of control group (128.8 ± 35.4 vs 144.1 ± 40.9 min, *P* = 0.006). There were no significant differences in postoperative hospital stay, VAS score on first postoperative day, incidence of seroma and hematoma, urinary retention and other complications (*P* > 0.05). None of the patients occured hernia recurrence, mesh infection, intestinal obstruction and other complications.

**Conclusions:**

The right-side approach to enter Retzius space is safe and feasible in TAPP surgery of bilateral inguinal hernia. Compared with the conventional approach, it can shorten the operative time and has certain advantages.

**Supplementary Information:**

The online version contains supplementary material available at 10.1186/s12893-023-01917-8.

## Background

Inguinal hernia repair is the most common operation in general surgery [1], more than 20 million inguinal hernia repairs are performed worldwide each year [2]. In recent years, with the development of the materials science and laparoscopic technology, as well as the in-depth research on the anatomy of the inguinal region, the surgical methods of inguinal hernia repair have been continuously improved, as innovated from traditional Bassini repair to Lichtenstein tension-free repair to laparoscopic repair. Laparoscopic transabdominal preperitoneal inguinal hernia repair (TAPP) is more favored by patients and clinicians because of slight pain, quick recovery and less invasion [3, 4]. Moreover, TAPP has a wide range of applications and can be applied to all kinds of inguinal hernia, but large irreducible hernias, hernias in patients after previous major lower abdominal surgery or hernias in patients who not fit for general anesthesia are limiting factors for TAPP [5]. Bilateral inguinal hernia repair occupies a certain proportion in inguinal hernia surgery. Nicolas et al. showed that the incidence of bilateral inguinal hernia reached 30% [6], laparoscopic repair is recommended for bilateral inguinal hernia according to International Guide to Inguinal Hernia 2018 [7]. Obviously, bilateral cases take longer and cost more than one side [6], what’s more, bilateral inguinal hernia repair is not a simple superposition of left and right hernia repair, but has its own operational difficulties. In this study, we compared the application effect of two surgical techniques to enter Retzius space in TAPP surgery for bilateral inguinal hernia, one was a new approach which was enter through right side, another was conventional path, to find a more time-saving, labor-saving and safe method for bilateral inguinal hernia.

## Methods

### Study design and participants

Retrospective analysis was performed on 189 patients who were diagnosed with bilateral inguinal hernia preoperatively or intraoperatively and underwent selective TAPP in the general surgery department of Shaanxi Provincial People's Hospital from January 2015 to September 2020. Inclusion criteria were ≥ 18 years, preoperative or intraoperative diagnosis of bilateral inguinal hernia. Exclusion criteria were: (1) recurrent hernia, sliding hernia; (2) strangulated hernia; (3) ascites, connective tissue disease, heart or renal failure; (4) taking drugs that affect blood coagulation; (5) patients who cannot tolerate general anesthesia; and (6) Mental cognitive dysfunction, audiovisual dysfunction and other disorders can’t participate in the clinical researchers. Bilateral TAPP was performed in 94 patients using the right-side approach (research group), and in 95 patients with conventional approach (control group). The comparison of general conditions between the two groups were shown in Table 1, and there was no significant difference in two groups (*P* > 0.05). All the surgeries were performed by the same team of surgeons. The surgeon was a 7-years of experience in laparoscopic hernia repair specialist, who has performed almost 500 TAPP, and the laparoscope holder was a qualified surgeon (Raw data was shown in Additional file 1).

**Table 1:** Comparison of demographic properties

	Research group	Control group	Statistical value	*P* value
Age (years)^a^	62.5 ± 15.3	61.7 ± 15.1	*t* = 0.398	0.691
Male/female	85/9	83/12	*χ*^*2*^ = 0.447	0.504
BMI (Kg/m^2^)	23.5 (22.6,23.9)	23.2 (22.7,23.8)	*z* = − 0.804	0.421
Type of hernia			*χ*^*2*^ = 4.435	0.218
Indirect	113	105		
Direct	57	74		
Femoral	5	4		
Multiple	13	7		
Maximum diameter of hernia sac (cm)^b^	4.0 (3.0,5.5)	4.0 (3.0,5.0)	*z* = − 0.098	0.922

^a^Values are presented as mean ± SD

^b^The maximum diameter of hernia sac is the average of the maximum diameter of bilateral hernia sac

### Surgical technique

All patients were performed under general anesthesia, and a 1 cm incision was made above the umbilicus as the observation hole. Made one 0.5 cm incision at each side of the lateral edge of rectus abdominis at the level of umbilicus as operation hole, established CO_2_ pneumoperitoneum and maintain pneumoperitoneum pressure at 12–15 mmHg.

#### Right-side approach group (research group)

**Fig. 1:**
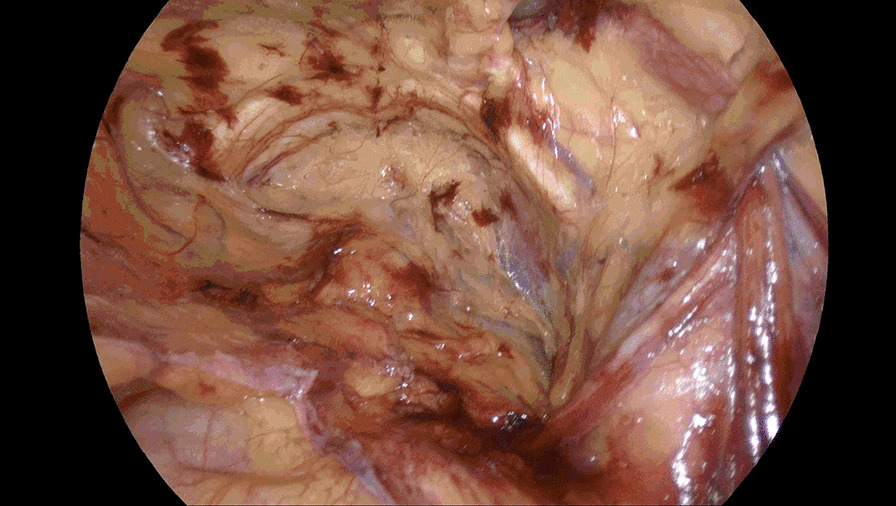
Dissociate right Retzius space medially to left beyond the symphysis pubis approximately 5 cm

**Fig. 2:**
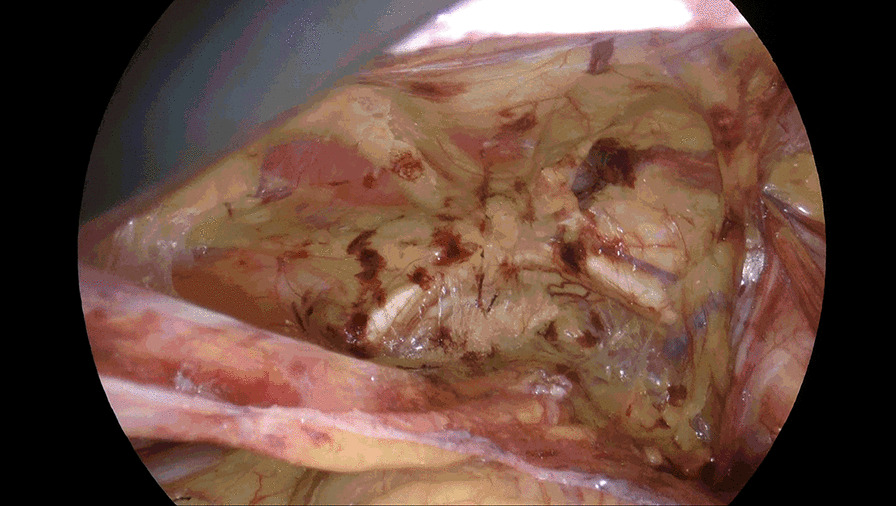
Enlarge right Retzius space

**Fig. 3:**
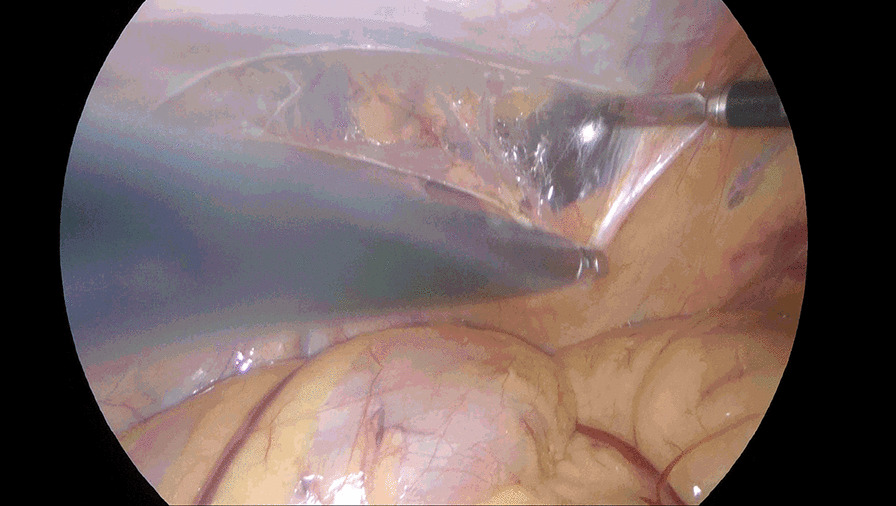
Only a single layer left after previous separation from right to left

**Fig. 4:**
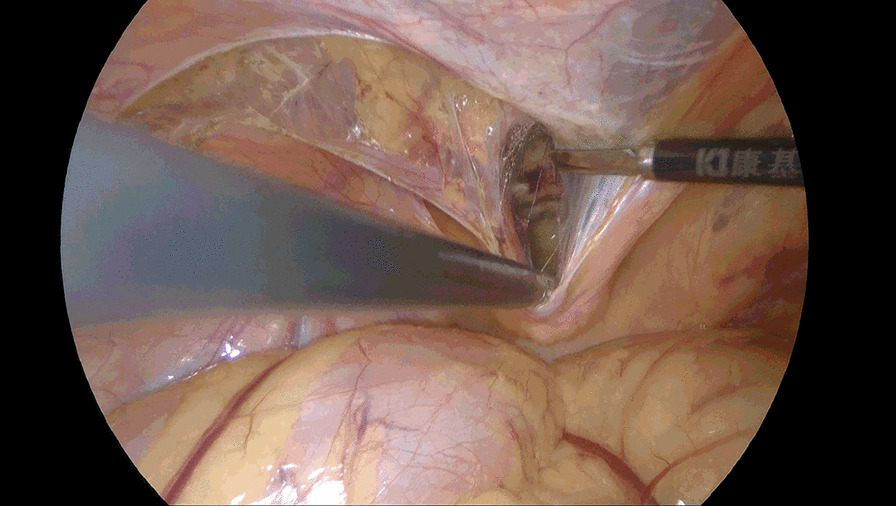
Separate the single layer and arrive at the left Retzius space and expose left pectineal ligament

Incise the peritoneum in the right inguinal region 2 cm above the hernia defect, bordered by anterior superior iliac spine laterally and medial umbilical fold medially. Care should be taken to protect the inferior epigastric vessels. Dissociate the preperitoneal space completely. Dissociate Retzius space medially to the symphysis pubis, further dissociate to the left beyond the symphysis pubis approximately 5 cm (Fig. 1) or the left Corona mortis is faintly visible, instead of being separated to the left of the symphysis pubis 1–2 cm as conventional approach. Take this as boundary and continue to enlarge the Retzius space upward and downward form right side (Fig. 2), bordered by 2 cm below the pectineal ligament inferiorly, 4 cm above the right hernia ring superiorly, laterally to the anterior superior iliac spine. Dissect the spermaduct and spermatic vessels off of the peritoneum, dissociate hernia sac, reset or sever the hernial sac. Incise the peritoneum in the left inguinal region, only a single layer left after previous separation from right to left (Fig. 3), separate the single layer and arrive at the left Retzius space and expose left pectineal ligament (Fig. 4), remain dissociations are the same as right. After complete the dissociation of both sides, two standard 10 × 15 cm polypropylene meshes are placed in bilateral preperitoneal space, cover Myopectineal orifice. Spread out and fix the mesh. Suture peritoneum and abdominal wall incisions.

#### Conventional approach group (control group)

The Retzius space is first dissociated medially from right to about 1–2 cm beyond the symphysis on the contralateral side, then dissociated medially from left until penetrating the preperitoneal space and the remaining surgical procedures were the same as those in the research group.

### Observed indicator

Operative time, postoperative hospital stay, Visual Analogue Scale (VAS) score on first postoperative day, postoperative seroma and hematoma, hernia recurrence, mesh infection, postoperative pain, urinary retention, intestinal obstruction and other complications were recorded in each group.

### Statistical analysis

SPSS 24.0 software was used for statistical analysis. The continuous variables conforming to normal distribution were reported as Mean ± SD, data were analyzed by two-samples *t*-test. Non-normally distributed continuous variables were reported by M (Q1, Q3), data were analyzed by Mann–Whitney *U*-test. The categorical variables were reported as percentage and were analyzed by Chi-square test. *P* < 0.05 was considered statistically significant.

## Results

All patients completed surgery successfully, and completed follow-up, the follow-up period was 2–70 months, the median follow-up was 34 months. Patients were followed up in outpatient department within 1 year and by telephone for more than 1 year, and all follow-up was completed by 31 November 2020.

### Comparison of demographic properties

The demographic properties of patients in the two groups included gender, age, Body Mass Index (BMI), hernia type, and the maximum diameter (cm) of the hernia sac (Table 1), and there were no significant differences between two groups(*P* > 0.05).

### Comparison of operative time

The operative time of the research group was significantly shorter than that of the control group (128.8 ± 35.4 min vs 144.1 ± 40.9 min, *P* = 0.006) (Table 2).

**Table 2:** Operative outcomes

	Research group	Control group	Statistical value	*P* value
Operative time (min)^a^	128.8 ± 35.4	144.1 ± 40.9	*t* = − 2.755	0.006
VAS score^b^	2.0 (1.0,2.0)	2.0 (2.0,3.0)	*z* = − 1.523	0.128
Postoperative hospital stay (day)	2.0 (2.0,3.0)	2.0 (2.0,2.0)	*z* = − 1.428	0.153
Seroma/hematoma (case)	8 (8.511%)	9 (9.474%)	*χ*^*2*^ = 0.054	0.817
Urinary retention (case)	2 (2.128%)	3 (3.158%)	*χ*^*2*^ = 0	1.000
Other complications (case)	1 (1.064%)	1 (1.053%)	*χ*^*2*^ = 0	1.000

^a^Values are presented as mean ± SD

^b^Visual Analogue Scale(VAS) score on first postoperative day

### Comparison of postoperative conditions

None of patients had recurrence of hernia, mesh infection and intestinal obstruction during follow-up time. There was no significant difference in postoperative hospital stay and VAS score on first postoperative day (*P* = 0.153, *P* = 0.128, respectively). The incidence of seroma and hematoma between the two groups has no significant difference (8 vs 9, *P* = 0.817), and all 17 patients recovered spontaneously without any intervention. Two cases in research group and 3 cases in control group experienced postoperative urinary retention, indwelled catheter and removed successfully before discharge, they were followed up regularly in urology department, and there was no statistically significant difference between the two groups (*P* = 1) (Table 2).

## Discussion

The Myopectineal orifice (MPO) is first proposed by Dr. Henri Fruchaud in 1956 as an obvious weakness of the pelvis region, and is considered to have become weakened during the evolutionary process as humans became bipedal, stood [8]. MPO is a key unifying anatomical structure, which is up to the conjoined tendon and down to Cooper ligament, inward to the rectus abdominus muscle and rectus sheath and outward to the iliopsoas muscle, encompassing Hesselbach’s triangle, the deep inguinal ring, and the femoral canal. It is susceptible to hernia because this area is made up of aponeurotic tissue and lacks of muscle coverage [9]. Stoppa is an early advocate of placing a giant mesh in the anterior peritoneal space to inguinal hernia repair by covering the MPO [10]. Professor Stoppa's "giant prosthesis" technique is particularly for treatment of complex, giant or recurrent inguinal hernia compared with the conventional approach [11]. However, the classical Stoppa technique requires a large incision in the midline of hypogastrium, which may cause postoperative pain and incision infection [12]. In 1992, Maurice Arregui first reported the technique of TAPP [13] which combined the experience of Stoppa with the concept of minimally invasive to repair an inguinal hernia. It preserves intact anatomy of the weak area of the pelvic cavity as well as places a large mesh, what’s more, it's more minimally invasive without large incision in hypogastrium. TAPP surgery is performed by dissecting MPO and placing a mesh to cover MPO in the preperitoneal space to repair the inguinal hernia comprehensively, including repair of the indirect hernia, direct hernia and femoral hernia.

Dissection of the Retzius space, also known as the retropubic space, is essential during TAPP surgery. Divide the preperitoneal space to midline can enter the Retzius space and expose symphysis pubis. In bilateral repair inguinal hernia, dissect Retzius space completely is indispensable. Through this space, the meshes covering the MPO on both sides are partially overlapped in the midline, repairing more efficiently. Stoppa technique and Retzius space, which connecting the bilateral MPO in the preperitoneal space, provide the theory foundation for this study.

Generally, in TAPP surgery for bilateral inguinal hernia, the Retzius space is first dissociated medially to about 1 cm beyond the symphysis on the contralateral side [14], then dissociate medially from another side until penetrating the preperitoneal space. In this study, the right side is first dissociated to the left beyond the symphysis pubis approximately 5 cm instead of 1 cm, and dissect a single layer from left can complete penetration easily. Why we choose 5 cm instead of further, since further dissociation might injury the left Corona mortis, which is about 6 cm away from the symphysis pubis [15]. Corona mortis is vascular connection of Obturator Vessels and Inferior Epigastric Vessels [16, 17], adjacent to the pectineal ligament. Once Corona mortis is injured, the broken end of the vessel retracts to the pelvic cavity, causing uncontrolled bleeding even fatal bleeding [18]. According to Rusu [19], Corona mortis are classified into three types: arterial type, venous type and mixed type. The incidences of Corona mortis are different reported by various researchers. Lau and Lee [20] found that the incidence was 40% in the dissection of retropubic vessels of patients who undergoing laparoscopic inguinal hernia repair, while 52% was reported by Pellegrino in 25 patients who undergoing gynecological tumor resection by laparoscopy [21]. Corona mortis do exist, although its incidence varies. Corona mortis injury resulting in bleeding is troublesome, so prevent the injury is of technical importance. Based on the review of literature [15] and our intraoperative experience, the author believe that it is safe to dissociate the Retzius space from the right side to about 5 cm beyond the symphysis on the contralateral side, without damaging of Corona mortis.

In terms of operative time, the right-side approach takes less time than the conventional approach, the author detailed analyses reasons as follow: Firstly, in conventional approach, it is inconvenient for most dominant right hands to dissociate the left Retzius space. What’s more, after entering Retzius space, the left hand needs to push the urinary bladder away and the right hand dissociate crosswise, so the two hands interfere with each other. While in right-side approach, the surgeon uses right hand to dissociate Retzius space from right and operate in the preperitoneal space in most of the operation, the surgical direction is consistent with the characteristics of most surgeons using the right dominant hand, thus reducing the difficulty of operation, shorten operation time. Secondly, the pectineal ligament always serves as a definite anatomic landmark to guide the efficient dissociation of Retzius space. In right-side approach, we dissociate Retzius space from right to the left beyond the symphysis pubis 5 cm along the pectineal ligament, then separate only a single layer from the left side can complete penetration easily, unlike conventional approach, which need to dissect the pectineal ligament on both sides resulting in longer operation time. Thirdly, laparoscope holder act as the eyes of the surgeon in TAPP, good coordination between the surgeon and the “eyes” also shorten the operation time. In right-side approach, laparoscope holder uses 30° laparoscope and stay in the same direction with the surgeon at most operative time, make the image clear and mutual interference between laparoscope and surgical instruments less. However, when the left Retzius space is dissociated by conventional approach, the laparoscope should always turn to lower left, it is extremely easy to collide with surgical instruments, thus increasing the difficulty of operation.

In terms of complications, there are no adverse events such as hernia recurrence, chronic pain, mesh infection, puncture hole hernia and intestinal obstruction in both right-side approach and conventional approach, indicate that right-side approach has no affection on the incidence of surgical complications.

The right-side approach to dissociate Retzius space can effectively shorten the operation time without increasing the incidence of complications. In application of this approach, the author summarizes the following cautions: First, dissociate and enter the right Retzius space should be the first step regardless of the hernia size, and bilateral mesh placement should be performed after the dissociation of bilateral preperitoneal space is all completed, if not, the right mesh may displace due to unintentional touch during dissociation of the left area. What’s more, sometimes the dissociation from right can’t reach 5 cm to the left of the pubic symphysis, because they will encounter the left hernia sac before reaching 5 cm in some thin patients with straight hernia or femoral hernia. We suggest that dissociation should alter to from left instead, for it is relatively easier and less likely to cause intraoperative concomitant injury. Last, the left inferior epigastric vessels may be seen when dissociate from the right inguinal region by this method if the left indirect hernia sac is too small. The surgeon must always be aware of the presence of these vessels. On the one hand, you should be careful to avoid damaging them, on the other hand, don't take them for the vessels of the bladder or rectus abdominis, or you could enter to the wrong layer. If the anatomy is indeed unclear, it is recommended to discontinue the operation from the right side and to dissociate from the left to avoid unnecessary injury. TAPP repair of bilateral inguinal hernia should be considered as a collective concept, rather than as an ill-considered superposition of left and right. The Retzius space, as a common space for bilateral inguinal hernia, is a bridge between the two sides of the inguinal area. In this study, it connects the "bridge" that the new approach can be realized.

## Conclusion

In summary, the right-side approach is a safe and effective method in TAPP surgery of bilateral inguinal hernia, which technically improve operations, effectively shorten the operative time without increasing the incidence of complications. Since this study is a retrospective cohort study, there may be selection bias, which requires further verification by prospective large sample study.

## Supplementary Information

**Additional file 1:** The raw data.

## Data Availability

The datasets used and analyzed during the current study are available from the corresponding author on reasonable request.

## Abbreviations

VAS: Visual analogue scale
BMI: Body mass index
MPO: Myopectineal orifice
TAPP: Transabdominal preperitoneal inguinal hernia repair
